# Cardiovascular effects of urocortin 2 and urocortin 3 in patients with chronic heart failure

**DOI:** 10.1111/bcp.13033

**Published:** 2016-07-28

**Authors:** Colin G. Stirrat, Sowmya Venkatasubramanian, Tania Pawade, Andrew J. Mitchell, Anoop S. Shah, Ninian N. Lang, David E. Newby

**Affiliations:** ^1^ British Heart Foundation/University Centre for Cardiovascular ScienceUniversity of EdinburghEdinburghUK; ^2^ British Heart Foundation Glasgow Cardiovascular Research Centre, Institute of Cardiovascular and Medical SciencesUniversity of GlasgowGlasgowUK

**Keywords:** cardiac, heart failure, inotrope, urocortin, vasodilator

## Abstract

**Aims:**

Urocortin 2 and urocortin 3 may play a role in the pathophysiology of heart failure and are emerging therapeutic targets. We aimed to examine the local and systemic cardiovascular effects of urocortin 2 and urocortin 3 in healthy subjects and patients with heart failure.

**Methods:**

Patients with heart failure (*n* = 8) and age and gender‐matched healthy subjects (*n* = 8) underwent bilateral forearm arterial blood flow measurement using forearm venous occlusion plethysmography during intra‐arterial infusions of urocortin 2 (3.6–36 pmol min^−1^), urocortin 3 (360–3600 pmol min^−1^) and substance P (2–8 pmol min^−1^). Heart failure patients (*n* = 9) and healthy subjects (*n* = 7) underwent non‐invasive impedance cardiography during incremental intravenous infusions of sodium nitroprusside (573–5730 pmol kg^−1^ min^−1^ ), urocortin 2 (36–360 pmol min^−1^ ), urocortin 3 (1.2–12 nmol min^−1^) and saline placebo.

**Results:**

Urocortin 2, urocortin 3 and substance P induced dose‐dependent forearm arterial vasodilatation in both groups (*P* < 0.05 for both) with no difference in magnitude of vasodilatation between patients and healthy subjects. During systemic intravenous infusions, urocortin 3 increased heart rate and cardiac index and reduced mean arterial pressure and peripheral vascular resistance index in both groups (*P* < 0.01 for all). Urocortin 2 produced similar responses to urocortin 3, although increases in cardiac index and heart rate were only significant in heart failure (*P* < 0.05) and healthy subjects (*P* < 0.001), respectively.

**Conclusion:**

Urocortins 2 and 3 cause vasodilatation, reduce peripheral vascular resistance and increase cardiac output in both health and disease. These data provide further evidence to suggest that urocortins 2 and 3 continue to hold promise for the treatment of heart failure.

## What is Already Known about this Subject

- Urocortins 2 and 3 are emerging therapies for treating heart failure.
- Urocortins 2 and 3 reduce peripheral vascular resistance and increase cardiac output in both health and disease.

## What this Study Adds

- This is the first direct head‐to‐head comparison of urocortin 2 and urocortin 3 in man.
- These data provide further evidence that urocortin 2 and urocortin 3 hold major potential for the treatment of heart failure.

## Introduction

There are almost six million people living with heart failure in the USA. This comes at an annual cost to the US economy of over $30 billion 1. Despite many evidence‐based therapies for patients with chronic heart failure, treatments for acute heart failure are limited, less well developed and have not been shown to improve clinical outcomes. Indeed the use of inotropic agents has been associated with harm 2, 3, 4. However, the tentative but promising improvements in clinical outcome seen with serelaxin in the recent RELAX‐AHF study 5 has renewed enthusiasm for the assessment of novel vasoactive mediators in this important patient group.

The urocortins are an endogenous peptidic hormone group comprising urocortin 1, urocortin 2 and urocortin 3 with vasodilator, inotropic and lusitropic effects 6. Urocortins 2 and 3 counteract many of the effects of corticotrophin‐releasing hormone (CRH) 7, 8, 9, 10 and act on the CRH‐receptor 2 (CRH‐R2), a G‐protein coupled receptor that is expressed abundantly in the heart and peripheral vasculature 11, 12. Urocortin 2 causes vasodilatation in healthy volunteers, augments cardiac output and reduces vascular resistance in healthy humans, patients with acute decompensated, and also chronic stable, heart failure 13, 14, 15, 16.

A recombinant acetate salt of urocortin 3 (JNJ‐39 588 146 [recombinant stresscopin]), improved cardiac output whilst reducing vascular resistance in a multicentre study of patients with chronic stable heart failure 17. We recently demonstrated that urocortins 2 and 3 increase forearm blood flow in young healthy volunteers 15. To date, no clinical study has separated the regional from systemic effects of urocortins 2 and 3 in heart failure or conducted a direct head to head comparison of their effects.

We aimed to evaluate and compare the local and systemic cardiovascular effects of urocortins 2 and 3 in patients with heart failure and healthy subjects by assessing (i) local forearm arterial blood flow using venous occlusion plethysmography and (ii) cardiac output and vascular resistance using thoracic bioimpedance cardiography. We hypothesized that urocortins 2 and 3 would cause vasodilatation, reduced peripheral vascular resistance and increased cardiac output in both healthy subjects and patients with heart failure.

## Methods

Both studies were approved by the local research ethics committee (South East Scotland REC 01) and carried out in accordance with the Declaration of Helsinki. The registered clinical trials on UKCRN were ID 10749 and 13002.

Written informed consent was obtained from all participants prior to the study.

### Study participants

Patients with heart failure were eligible if they were aged 18–80 years, New York Heart Association (NYHA) symptom class II–III and had echocardiographically confirmed left ventricular ejection fraction <35% with left ventricular end diastolic diameter > 5.5 cm. Patients were required to be receiving maximally tolerated doses of angiotensin‐converting enzyme inhibitor and β‐adrenoceptor blocker therapies for at least 3 months. Healthy subjects had no significant previous medical history and were on no regular medications. Exclusion criteria for both groups included systolic blood pressure > 190 mmHg or <90 mmHg, haemodynamically significant valvular heart disease and other severe or significant co‐morbidities including bleeding diathesis, renal or hepatic failure, anaemia or recent infective/inflammatory conditions. Women of child bearing potential were also excluded.

### Protocol A: local vascular study

**Figure 1:**
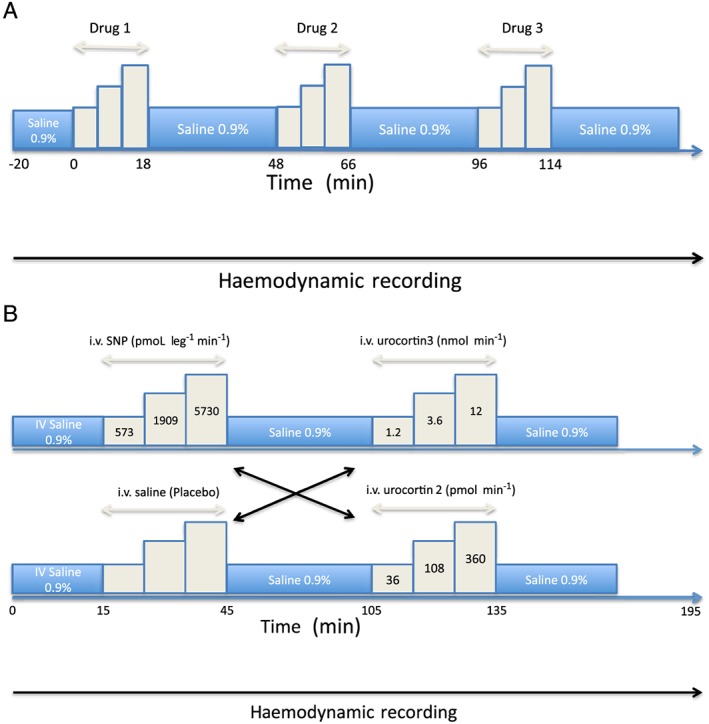
A) Schematic representation of study protocol A – vascular study and B) schematic representation of study protocol B – systemic study. A) forearm blood flow. B) systemic haemodynamic responses

This was a randomized study (Figure 1A) of eight patients with heart failure and eight age and gender‐matched healthy subjects. Subjects attended once each to receive incremental intra‐arterial infusions of urocortin 2 (3.6, 12, 36 pmol min^−1^, molecular weight 4450.3 g mol^−1^), urocortin 3 (360, 1200, 3600 pmol min^−1^, molecular weight 4137.9 g mol^−1^) and substance P (2, 4, 8 pmol min^−1^ [control endothelium‐dependent vasodilator]) for 6 min at each dose with a 30 min washout period between agents.

Forearm venous occlusion plethysmography studies were performed with the subject lying supine, in a quiet, temperature‐controlled room (22–25°C). Participants fasted for 4 h prior to the study and refrained from alcohol and caffeine for 24 h prior to the study. Venous cannulae (17G) were inserted into large subcutaneous veins in the antecubital fossae of both arms at the start of the study to facilitate periodic venous sampling. Heart rate and blood pressure were monitored at regular intervals throughout the study with a semi‐automated oscillometric sphygmomanometer (Omron 705IT). Participants underwent brachial artery cannulation of the non‐dominant forearm with a 27 standard wire‐gauge steel needle for agent infusion. Forearm blood flow was measured in the infused and non‐infused forearms using bilateral venous occlusion plethysmography as described previously 18, 19.

### Protocol B: systemic study

This was a randomized, double‐blind, placebo controlled crossover study (Figure 1B). Three patients with heart failure and three volunteers participants were involved in both studies. Patients with heart failure (*n* = 9) and healthy subjects (*n* = 7) were recruited. Participants had venous cannulae inserted into both antecubital fossae and attended on two occasions, receiving intravenous infusions of saline (placebo) or SNP (573, 1909, 5730 pmol kg^−1^ min^−1^) followed by either urocortin 2 (36, 108, 360 pmol min^−1^) or urocortin 3 (1.2, 3.6, 12 nmol min^−1^). On the second visit, participants received the two remaining agents not administered on visit 1. Each agent was given in three ascending doses for 10 min at each dose. A 1 h saline washout was given between agents and a further 30 min of saline washout was administered after the cessation of the second agent.

#### Haemodynamic monitoring

Haemodynamic measurements were recorded throughout the study. Cardiac output, blood pressure and stroke volume were recorded using non‐invasive thoracic impedance cardiography (NCCOM3‐R7, BioMed, CA, USA or Cardioscreen 1000, Medis, Germany) and oscillometric sphygmomanometry (Omron HEM‐705CP, Omron, Matsusaka, Japan). Values were indexed to body surface area where appropriate and peripheral vascular resistance index was calculated using recorded measurements (PVRI = MAP/CI).

#### Safety

Study stopping criteria were in place to ensure participant safety. Criteria included a drop in diastolic blood pressure of >25 mmHg, fall in heart rate below 50 beats min^−1^ or rise above 120 beats min^−1^, at the request of the participant, attending nurse or the attending physician.

#### Venous sampling

Baseline blood samples were drawn for assessment of full blood count, renal function, glucose and cholesterol concentrations at the start of each study. The local clinical biochemistry and haematology reference laboratories performed analysis.

#### Data analysis and statistics

Data were collected in a double‐blind fashion for both studies. Data were analyzed, where appropriate, by analysis of variance (ANOVA, one way and two way with repeated measures where appropriate). All results in figures are expressed as mean ± SEM. All statistical analysis was performed with GraphPad Prism, version 6 (GraphPad Software, San Diego, CA, USA). Statistical significance was taken as two‐sided *P* < 0.05.

## Results

Patients with heart failure and healthy subjects recruited were predominantly male and middle‐aged. Heart failure patients had greater BMI compared with healthy subjects. Participant characteristics are shown in Table 1. Patients with heart failure were receiving maintenance heart failure therapy. Patients and volunteers were age and gender‐matched in protocol A and age matched alone in protocol B. Three patients with heart failure and three volunteer participants were recruited to both studies.

**Table 1:** Participant characteristics for protocol A and B

	**Protocol A**			**Protocol B**		
	**Heart failure patients (*n* = 8)**	**Healthy subjects (*n* = 8)**		**Heart failure patients (*n* = 9)**	**Healthy subjects (*n* = 7)**	
**Age (years)**	55.5 [52.25–67]	57 [53–65.75]	*P* = 0.84	58 [50–66.5]	58 [45–66]	*P* = 0.56
**BMI**	30 ± 5.4	25.1 ± 2.3	*P* = 0.03	32.2 ± 4.3	25.9 ± 1.9	*P* = 0.003
**Gender**	5 M, 3F	5 M, 3F		7 M, 2F	4 M, 3F	
**ACEi/ARB**	8 (100)	n/a		9 (100)	n/a	
**BB**	6 (75)	n/a		7 (78)	n/a	
**MRA**	5 (63)	n/a		8 (89)	n/a	
**Digoxin**	1 (13)	n/a		4 (44)	n/a	
**Loop diuretic**	6 (75)	n/a		6 (67)	n/a	
**Haemoglobin (visit 1 *vs* visit 2, g l^−1^)**	n/a	n/a		136.0 ± 14.46 *vs* 133.0 ± 14.96 (*P* > 0.99)	143.7 ± 7.111 *vs* 135.3 ± 8.524 (*P* > 0.99)	
**Creatinine (visit 1 *vs* visit 2, μmol l^−1^)**	n/a	n/a		109.8 ± 39.66 *vs* 116.3 ± 74.95 (*P* > 0.99)	75.29 ± 8.635 *vs* 72.67 ± 7.840 (*P* > 0.99)	

*n* (%), mean ± SD, median [interquartile range]

### Protocol A – vascular study

The intra‐brachial infusion of all three drugs was well tolerated with no adverse effects. Intra‐brachial infusion of urocortin 2 and 3 produced localized, self‐limiting forearm flushing and some facial flushing in both groups of participants as noted in previous studies 15.

**Figure 2:**
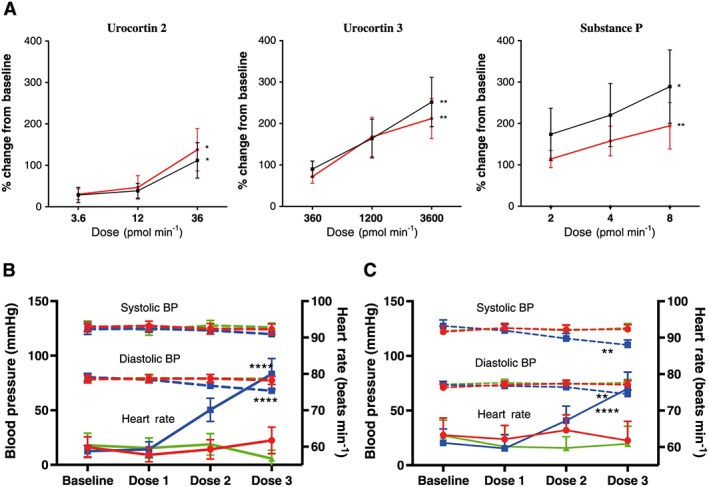
Haemodynamic responses during local administration of urocortin 2, urocortin 3 and substance P (protocol A). A) Percentage change in forearm arterial blood flow to urocortin 2 (3.6–36 pmol min^−1^), urocortin 3 (360–3600 pmol min^−1^) and substance P (2–8 pmol min^−1^) in patients with heart failure (red) and in healthy subjects (black). B) Non‐invasive systemic hemodynamic responses to intra‐arterial urocortin 2 (red), urocortin 3 (blue) and substance P (green) in healthy subjects (A) and patients with heart failure (B). (**** = *P*,0.0001,** = *P* < 0.01, * = *P* < 0.05)

Urocortin 2, urocortin 3 and substance P all evoked dose‐dependent forearm arterial vasodilatation in both participant groups (mean changes across the three doses [95% CI] from baseline as follows: urocortin 2 +60% [9–111] *P* < 0.05, +72% [21–123] *P* < 0.01; urocortin 3 +167% [100–237] *P* < 0.0001, +151% [82–219] *P* < 0.0001; substance P +227% [130–326] *P* < 0.0001, +155% [57–253] *P* < 0.001 for healthy controls and heart failure patients, respectively; Figure 2A). There were no significant differences in changes in forearm blood flow between heart failure patients and healthy subjects (urocortin 2 +12% [−40 to 63%] *P* = 0.84, urocortin 3 −18% [−86 to +50%], *P* = 0.80; substance P −72% [−170 − +26%] *P* + 0.19).

Blood pressure and heart rate remained unchanged in both groups in response to urocortin 2 and substance P. At the highest dose, urocortin 3 induced a transient tachycardia compared with baseline in both groups (heart failure +14.9 beats min^−1^ [20.9 to 8.9], healthy volunteers +21.3 mmHg [27.3 to 15.3] both *P* < 0.0001) that was accompanied by a drop in systolic (−17.4 mmHg [−7.9 to −26.9], *P* < 0.0001) and diastolic blood pressure in patients with heart failure (−8.4 mmHg [−3.5 to −13.3], *P* < 0.001) and a drop in diastolic blood pressure alone in healthy subjects (−12.4 mmHg [−7.5 to −17.3], *P* < 0.0001, Figure 2B). Non‐infused forearm blood flow remained unchanged throughout the study (data not shown).

### Protocol B – systemic study

Participants displayed hypotension at the higher doses of SNP initially administered in the study (17.2 nmol kg^−1^ min^−1^), frequently reaching study stopping criteria. A dose reduction to SNP (maximum dose of 5730 pmol kg^−1^ min^−1^) was made after two healthy subjects completed the study. For the urocortins, hypotension reaching study stopping criteria was met in one heart failure patient for urocortin 2 and two heart failure patients and one healthy subject for urocortin 3. Participants described dose‐dependent symptoms of tachycardia and a warm sensation with both urocortins, but there were no significant adverse events attributable to either urocortin 2 or urocortin 3. Full blood count and serum biochemistry was unchanged between the two study visits (Table 1).

**Figure 3:**
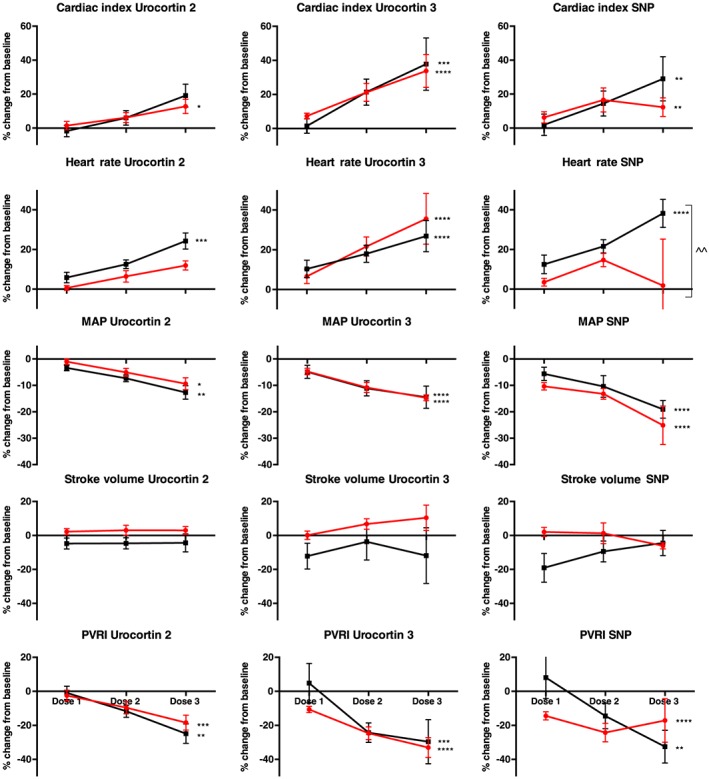
Changes in haemodynamic responses from baseline following systemic infusion (protocol B). Haemodynamic responses to infusions of urocortin 2, urocortin 3 and SNP in healthy subjects (black) and patients with heart failure (red) at doses 1–3 (D1–D3). * represents significant differences from saline placebo (not shown) across the three doses (see Table 2). ^ represents significant differences between participant groups. (****P < 0.0001, ***P < 0.001, **P < 0.01, *P < 0.05; ^^*P* < 0.01)

Urocortin 2 had no significant effect on cardiac index in healthy subjects and heart rate in heart failure patients when compared with saline placebo (*P* > 0.05 for both, see Figure 3 and Table 2 for results). Otherwise urocortin 2 and urocortin 3 increased cardiac index and heart rate and reduced mean arterial pressure and peripheral vascular resistance index in both patients with heart failure and healthy subjects (*P* < 0.05 for all). There was no effect of either urocortin 2 or urocortin 3 on stroke volume (*P* > 0.05 for both).

**Table 2:** Results of systemic infusion of urocortin 2 and 3

	Urocortin 2	Urocortin 3	SNP
**Healthy volunteers**			
**Cardiac index**	+11.9 [−3.1, +26.9] *P* = 0.17	+25.4 [+9.8, +41.0]***	+21.7 [+5.5, +37.8]**
**Heart rate**	+17.2 [+7.2, +27.1]***	+25.0 [+14.7, +35.4]****	+29.0 [+18.4, +39.8]****
**MAP**	−8.0 [−2.6, −13.5]**	−10.8 [−5.1, −16.4]****	−13.0 [−7.2, −18.8]****
**SV**	−3.7 [−13.8, +6.4] *P* = 0.77	−6.3 [−16.9, +4.2] *P* = 0.40	−6.8 [−17.4, +3.9] *P* = 0.35
**PVRI**	−17.6 [−3.5, −31.7]**	−23.1 [−8.4, −37.8]***	−22.1 [−6.9, −37.3]**
**Heart failure**			
**Cardiac index**	+10.1 [+1.0, +19.3]*	+23.5 [+14.3, +32.7]****	+14.2 [+3.8, +24.7]**
**Heart rate**	+7.1 [−0.8, +15.0] *P* = 0.1	+18.1 [+10.0 , +26.3]****	+7.4 [−1.8, +16.6] *P* = 0.16
**MAP**	−4.8 [−0.5, −9.0]*	−9.1 [−4.8, −13.5]****	−13.6 [−8.7, −18.5]****
**SV**	+2.7 [−2.4, +7.8] *P* = 0.51	+4.3 [−0.9, +9.5] *P* = 0.14	+0.7 [−4.8, +6.3] *P* = 0.99
**PVRI**	−14.0 [−5.3, −22.7]***	−25.2 [−16.2, −34.2]****	−22.1 [−12.2, −32.0]****

Mean difference [95% CI] of each agent with saline placebo across the three administered doses. * represents significant differences from placebo. (

**** *P* < 0.0001,

*** *P* < 0.001,

** *P* < 0.01,

* *P* < 0.05).

**Figure 4:**
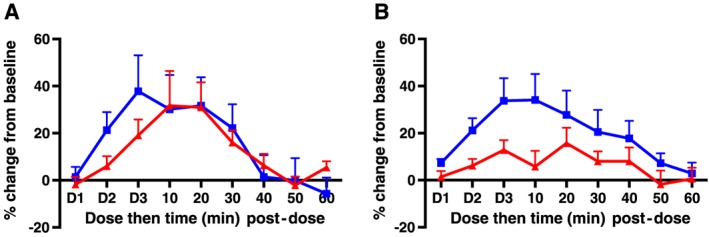
Duration of haemodynamic response (min) to intravenous urocortin 2 and urocortin 3 (protocol B). Effects of urocortin 2 (red) and urocortin 3 (blue) last 40–60 min after cessation of dose 3 (D3) before returning to baseline. At the doses used, urocortin 3 caused a greater increase in cardiac output compared with urocortin 2 in patients with heart failure (*P* < 0.0001) but not healthy subjects (*P* = 0.48)

At the doses used, urocortin 3 caused greater mean increases than urocortin 2 in cardiac index (+13.4 [+4.1 to +22.6], *P* < 0.01) and heart rate (+11.0 [+2.9 to +19.2], *P* < 0.01) and greater mean reductions in mean arterial pressure (+4.4 [0.0 to +8.7], *P* < 0.05) and peripheral vascular resistance index (+11.2 [2.2 to +20.1], *P* < 0.01) in patients with heart failure. No such haemodynamic differences existed between urocortin 2 and urocortin 3 in healthy subjects. No haemodynamic differences existed between participant groups for both urocortin 2 and urocortin 3 (*P* > 0.05 for all). Following cessation of the intravenous infusions, haemodynamic variables returned to baseline after 40–60 min (for example, cardiac index, Figure 4). Over the infusion and washout period, urocortin 3 again caused a greater increase in cardiac output (*P* < 0.0001) than urocortin 2 in patients with heart failure but not in healthy subjects (*P* = 0.48).

## Discussion

For the first time, we report the local vascular and systemic effects of urocortins in both patients with heart failure and healthy subjects. We demonstrate that both urocortin 2 and urocortin 3 increase cardiac index and reduce peripheral vascular resistance and their parenteral administration is feasible, safe and well tolerated. We conclude this haemodynamic profile suggests that urocortins 2 and 3 hold major potential for the treatment of acute heart failure.

Forearm venous occlusion plethysmography combined with intra‐arterial cannulation allows the assessment of local, sub‐systemic vasomotor effects of peptides without exerting a systemic response. This is particularly useful in the study of novel compounds, such as urocortin 2 and urocortin 3. We have previously demonstrated that urocortin 2 and 3 cause vasodilatation in healthy subjects and that is in part mediated by endothelium‐dependent factors such as nitric oxide and endothelium derived hyperpolarizing factor 15. Impaired endothelial function is a recognized feature and an independent predictor of adverse outcome in patients with heart failure 20, 21, 22, 23, 24. There is therefore a theoretical concern that urocortin would be less effective in patients with heart failure because of this concomitant endothelial dysfunction. However, we demonstrate that urocortin 2 and urocortin 3 evoked normal forearm arterial vasodilatory responses in our patients with heart failure. This suggests that either endothelial dysfunction does not have a meaningful impact on the actions of urocortin or our subjects did not have significant endothelial dysfunction. Interestingly, our patients did not demonstrate impaired substance P induced vasodilatation suggesting preserved endothelial function. This may reflect that the patients studied had stable heart failure symptoms and were well‐treated and receiving optimal medical therapy. Whether similar findings would be observed in patients with decompensated heart failure remains to be established.

During local intra‐arterial infusions of the urocortins, we were not anticipating observing any major changes in haemodynamic variables in either group. However, we found dose‐related increases in heart rate and falls in diastolic blood pressure, especially with urocortin 3 in healthy subjects. This suggests that at the doses used here, we had systemic spill over of urocortin such that we achieved vasoactive blood concentrations outwith the forearm. The doses where systemic systemic spill over was seen helped guide the dosing regime used in protocol B. We have seen systemic spill over previously with other compounds such as substance P 25 and this is heralded by systemic flushing and rises in the contralateral non‐infused forearm blood flow. However, here we observed no change in contralateral non‐infused forearm blood flow although subjects did develop some skin flushing. This suggests that other vascular beds, such as the dermal and splanchnic circulation, are more sensitive to the actions of the urocortins than the forearm circulation. The increase in heart rate at the highest dose of urocortin 3 was less pronounced in patients with heart failure and this was likely due to concomitant β‐adrenoceptor blocker therapy. Furthermore the associated lower systolic and diastolic blood pressures in this group may be explained by a relative lack of compensatory tachycardia response to vasodilatation in these patients.

During systemic intravenous infusions, we observed increases in cardiac output with both urocortins. This increase in cardiac output is likely to occur in response to the systemic vasodilatation induced by the urocortins. Although there was no change in stroke volume for either urocortin, this is not a direct measurement of inotropy and we cannot exclude a direct inotropic effect of urocortin on the heart, as previously seen with urocortin 2 in rodents 26. At the doses we administered, urocortin 3 exerted more marked haemodynamic effects than urocortin 2 in patients but not healthy subjects. This may reflect the differences in doses we employed, but may also reflect important differences between these two agents. This needs further exploration to determine which urocortin subtype is the most promising for clinical therapeutic development.

Differences in biological effects between the urocortins can be explained by the structural differences that exist between the two agents, generating conformational changes in the G protein coupled receptor, in turn altering secondary messenger systems that are responsible for creating the biological effects seen. However it may not be this simple and there may be other systems involved. Promising preclinical studies suggest a role for the urocortins in the inhibition of cardiac sympathetic nerve activity [SNA] 27, 28, often overactive in patients with heart failure. Although Ucn2 has been reported to increase skeletal muscle SNA in humans 29, this should not necessarily be seen as a discrepant finding as SNA responses typically are regionally differentiated.

Increases in heart rate have not been reported in recent studies with urocortin 2/stresscopin 16, 17. However higher doses of urocortin 2 14, comparable with the doses we have used here, saw similar increases in heart rate.

There may be concern regarding the clinical use of a heart failure therapy that is both positively chronotropic and inotropic. This combination of effects might predispose to increased myocardial oxygen consumption and potential arrhythmia, particularly in patients with coronary artery disease. We observed no episodes of arrhythmia or adverse effects in our study and, indeed, urocortin 2 has been shown to have anti‐arrhythmic effects that would be hugely beneficial in treating this group of patients 30, 31. However, our infusions were brief and adverse effects may be seen with longer infusions or in those patients with decompensated heart failure. In addition, avoidance of significant hypotension remains an important consideration, especially in patients with already low perfusion pressure or renal impairment. Results from our study suggest that dose titration during administration may be needed to optimize cardiac output and vasodilatory responses, whilst avoiding the unwanted effects of significant tachycardia or hypotension.

### Study limitations

This study included only patients with stable heart failure who were prescribed evidence‐based heart failure therapy that may have affected the response of these agents. However, there was no evidence of diminished effect with concomitant medical therapy in patients with heart failure. We did not include patients with acute decompensated heart failure but we did compare with healthy control subjects, not used in a similar sized study with urocortin 2 14. Although our results cannot be extrapolated to the setting of acute heart failure, there appears to be no reason why the beneficial effects seen in this study cannot be replicated in the acute heart failure setting provided an appropriate, controlled dosing regime is used. Furthermore studies using urocortin 2, and derivatives thereof, in the acute setting have already been carried out successfully. There does however remain a clear need for urocortin 3 to be trialled in this group of patients.

We observed increases in cardiac index in protocol B but it should be reinforced that this study was not designed to compare the relative contributions of chonotropy, inotropy and vasodilatation to the changes in cardiac index we recorded. Invasive studies would be required for this. Furthermore, the molar concentrations of urocortin 2 and urocortin 3 at each dose were different and differences in efficacy may reflect the differences in dose used and not true differences in potency.

Finally although there was no change in stroke volume for either agent, we did not conduct invasive haemodynamic monitoring which would be required for assessment of true inotropy and also useful for assessment of pulmonary capillary wedge pressure (PCWP). Surprisingly two recent studies did not detect a statistically significant reduction in PCWP in patients with heart failure with either urocortin 2/stresscopin 16, 17. However a clear trend toward a reduction in PCWP was seen in both studies.

In conclusion, we demonstrate that both urocortin 2 and urocortin 3 increase cardiac index and reduce peripheral vascular resistance. Their parenteral administration was feasible, safe and well tolerated. We conclude that these data provide further evidence suggesting urocortin 2 and urocortin 3 continue to hold promise for the treatment of heart failure.

## Competing Interests

All authors have completed the Unified Competing Interest form at www.icmje.org/coi_disclosure.pdf (available on request from the corresponding author) and declare no support from any organization for the submitted work, no financial relationships with any organizations that might have an interest in the submitted work in the previous 3 years and no other relationships or activities that could appear to have influenced the submitted work.
